# Elucidating the Possible Involvement of Maize Aquaporins and Arbuscular Mycorrhizal Symbiosis in the Plant Ammonium and Urea Transport under Drought Stress Conditions

**DOI:** 10.3390/plants9020148

**Published:** 2020-01-23

**Authors:** Gabriela Quiroga, Gorka Erice, Ricardo Aroca, Antonio Delgado-Huertas, Juan Manuel Ruiz-Lozano

**Affiliations:** 1 Departamento de Microbiología del Suelo y Sistemas Simbióticos, Estación Experimental del Zaidín (CSIC), Profesor Albareda nº 1, 18008 Granada, Spain; 2 Instituto Andaluz de Ciencias de la Tierra (IACT), CSIC-UGR, Armilla, 18100 Granada, Spain

**Keywords:** ammonium, aquaporin, arbuscular mycorrhizal symbiosis, N mobilization, urea

## Abstract

This study investigates the possible involvement of maize aquaporins which are regulated by arbuscular mycorrhizae (AM) in the transport in planta of ammonium and/or urea under well-watered and drought stress conditions. The study also aims to better understand the implication of the AM symbiosis in the uptake of urea and ammonium and its effect on plant physiology and performance under drought stress conditions. AM and non-AM maize plants were cultivated under three levels of urea or ammonium fertilization (0, 3 µM or 10 mM) and subjected or not to drought stress. Plant aquaporins and physiological responses to these treatments were analyzed. AM increased plant biomass in absence of N fertilization or under low urea/ ammonium fertilization, but no effect of the AM symbiosis was observed under high N supply. This effect was associated with reduced oxidative damage to lipids and increased N accumulation in plant tissues. High N fertilization with either ammonium or urea enhanced net photosynthesis (*A_N_*) and stomatal conductance (*gs*) in plants maintained under well-watered conditions, but 14 days after drought stress imposition these parameters declined in AM plants fertilized with high N doses. The aquaporin *ZmTIP1;1* was up-regulated by both urea and ammonium and could be transporting these two N forms in planta. The differential regulation of *ZmTIP4;1* and *ZmPIP2;4* with urea fertilization and of *ZmPIP2;4* with NH_4_^+^ supply suggests that these two aquaporins may also play a role in N mobilization in planta. At the same time, these aquaporins were also differentially regulated by the AM symbiosis, suggesting a possible role in the AM-mediated plant N homeostasis that deserves future studies.

## 1. Introduction

Nitrogen (N) is one of the most important macronutrients for plants, being required in significant amounts as is a constituent of nucleic acids, amino acids, proteins, lipids, co-enzymes, chlorophyll, phytohormones, and secondary metabolites [1]. However, the availability of this nutrient is very variable in soils, and losses in chemical fixation and leaching lead to deficiency in many agricultural lands, which at the end limit plant growth. Plants absorb N from soils mainly as nitrate (NO_3_^−^) or ammonium (NH_4_^+^). Nitrate is predominant in aerobic soils, while ammonium dominates in acidic soils or flooded wetlands [2]. NH_4_^+^ presence in soils depends on different factors (soil pH, accumulation of organic matter, temperature, soil oxygenation, presence of allelopathic substances, etc.). The augmentation of NH_4_^+^ deposition can also be the consequence of human activity, such as agriculture, and the increased levels of this nutrient can be toxic to plants [3].

On the other hand, urea (CH_4_N_2_O) is the most common N fertilizer because of its low cost, because it is also an important N metabolite. Its use in agriculture includes both foliar and soil applications, but water eutrophication is becoming a serious environmental problem by this practice. When applied to soils, urea is rapidly transformed by microorganism-derived urease into NH_4_^+^ and CO_2_ [2]. Nevertheless, the uptake of urea by plant roots was observed in independent experiments [4,5,6,7]. In fact, the assimilation pathways of NH_4_^+^ and urea were found to be very similar [4]. The characterization of high-affinity urea transporters, like ZmDUR3, confirmed the hypothesis of active urea uptake and transport in plants [8] Nevertheless, urea toxicity is still under debate, and similar effects to NH_4_^+^ were also reported [9].

Arbuscular mycorrhizal symbiosis plays important roles in nutrient acquisition by the majority of land plants. This symbiosis is mainly known by the enhanced uptake of phosphorous (P), but these fungi can also transfer N to their hosts, although their contribution is still under debate [10]. However, several studies suggest that P and N uptake and transport are tightly related in the AM symbiosis, and they could be controlling mycorrhizal functioning [11]. There are mainly evidence of ammonium and nitrate being taken up by the AM fungi, although they can also increase access to organic N forms from the soil [12,13]. However, the flow of N within the fungal hyphae would also involve the urea cycle, which produces urea and is further transformed in ammonium which would be transferred to the host plant [14]. LjAMT2;2, a plant high-affinity ammonium transporter was the first characterized to be involved in the uptake of N during arbuscular mycorrhizal symbiosis. It is localized in the periarbuscular membrane, and was only expressed in arbusculated cells of mycorrhizal roots, releasing ammonia in the cytoplasm of the root cells [15]. Several other ammonium transporter genes were identified in different plant species [16].

Recent evidence suggests that the affinity of the AM fungi for NH_4_^+^ uptake is five times higher than NH_4_^+^ uptake by plant systems, which means that the fungus may take this nutrient even under low N supply [11]. Moreover, it was demonstrated in mycorrhizal maize plants that the ability of the AM fungus to transfer N to the plant differs depending on the N source. In fact, ammonium was transferred at an equivalent rate as P, while nitrate seemed to not be transported [17]. AM fungi prefer the direct uptake of ammonium instead of nitrate due to the extra energy needed for the reduction of nitrate, required for the incorporation of N into organic compounds [16].

Apart from specific ammonium or urea transporters, plants contain an array of aquaporin isoforms (AQPs), which are ubiquitous membrane channels facilitating the passive flux of water and a range of small solutes [18]. Among the diversity of plant aquaporin substrates, urea and ammonium were found to be transported in heterologous systems [5,19,20]. Thus, it can be speculated that these proteins have a role in nitrogen mobilization in plants. In fact, differences in AQP gene expression or posttranslational modifications were found in different studies depending on N availability and form [21], being also suggested that the response is dependent on the plant variety [5]. In the case of maize, different aquaporins regulated by the AM symbiosis under drought stress were demonstrated to transport both N forms in yeast assays [22,23].

Herein, this study aims to investigate the possible involvement of AM-regulated aquaporins in the transport in planta of ammonium and/or urea under well-watered and drought stress conditions. Additionally, the study also attempted to better understand the implication of the AM symbiosis in the uptake of different N forms (urea and ammonium) and its effect on plant physiology and performance under drought stress conditions.

## 2. Results

**Figure 1:**
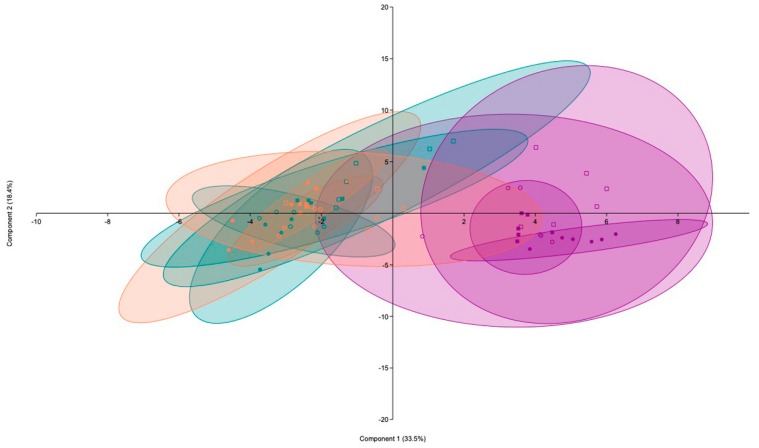
Principal components analysis (PCA) for data corresponding to the ammonium experiment. Plot with data run in PAST software, showing the two principal components (PC1 and PC2) which separated the samples by ammonium level. Blue color corresponds to no N treatments, orange color correspond to low N treatments and purple color to high N treatments. Solid circles represent non-AM plants under WW, solid squares represent non-AM plants under DS (during 14 days), empty circles represent AM plants under WW and empty squares represent AM plants under DS.

**Figure 2:**
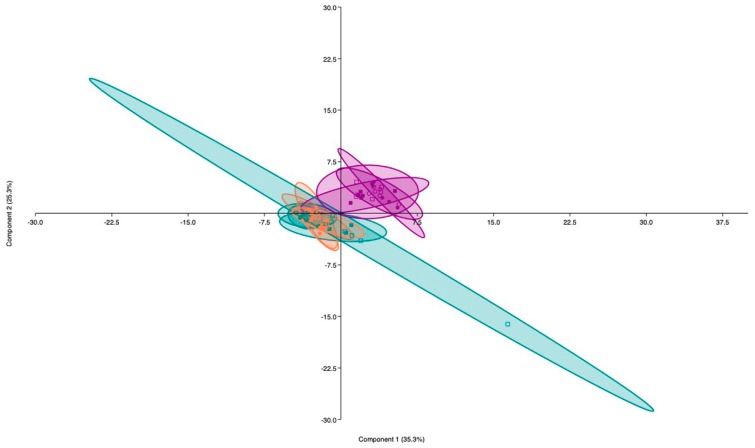
Principal components analysis (PCA) for data corresponding to the urea experiment. Plot with data run in PAST software, showing the two principal components (PC1 and PC2) which separated the samples by urea level. Blue color corresponds to no N treatments, orange color correspond to low N treatments and purple color to high N treatments. Solid circles represent non-AM plants under WW, solid squares represent non-AM plants under DS (during 14 days), empty circles represent AM plants under WW and empty squares represent AM plants under DS.

In this study, the principal components analysis (PCA) done for data obtained in the ammonium and urea experiments (Figure 1 and Figure 2) showed that axes PC1 and PC2 explained 51.9% of data variability in the ammonium experiment and 60.6% of data variability in the urea experiment. In both cases, the main factor affecting the data distribution is the N level applied. Thus, data of high N treatments distribute along the right side of vertical axis and data of no N and low N treatments distribute along the left side of this axis. Moreover, ANOVA results were included as supplementary material in Tables S1 and S2. 

### 2.1. Plant Growth and AM Fungal Colonization

In absence of N fertilization and under low N supply, mycorrhization enhanced plant fresh weight regardless of N form, both under well-watered (WW) and under drought stress (DS) conditions. However, under high N supply, mycorrhization did not have a significant effect on plant growth, with the exception of WW plants with high NH_4_^+^ supply, which slightly decreased plant fresh weight (Table 1).

At each watering regime, levels of root AM colonization were not significantly affected by the different N forms and concentrations applied (Table 1).

### 2.2. Oxidative Damage to Lipids and Chlorophyll Content

In NH_4_^+^-fertilized plants cultivated under WW conditions, mycorrhization reduced MDA production under low NH_4_^+^ level. Plants fertilized with high NH_4_^+^ exhibited a low MDA content, regardless of AM inoculation. Under DS conditions, mycorrhization reduced MDA accumulation both under low and no N supply. Again, plants fertilized with high NH_4_^+^ exhibited a low MDA content, regardless of AM inoculation. In urea-fertilized plants, mycorrhization did not affect the oxidative damage to lipids in WW plants. However, under such conditions, the plants that were not fertilized with any N presented higher MDA levels as compared to those fertilized with both urea levels (Table 1). Under DS, mycorrhization decreased MDA production in urea-fertilized plants and in those that did not receive any N. Moreover, MDA accumulation was also reduced with the highest urea concentration (Table 1).

Relative chlorophyll content was measured with SPAD by measuring dual optical absorbances. Values increased with high N-fertilization under both WW and DS conditions. In urea-fertilized plants under WW, mycorrhization slightly decreased chlorophyll levels compared to non-AM plants, while under drought stress it increased slightly the chlorophyll levels under low urea fertilization (Table 1).

### 2.3. Nitrogen and Carbon Concentrations in Tissues

**Figure 3:**
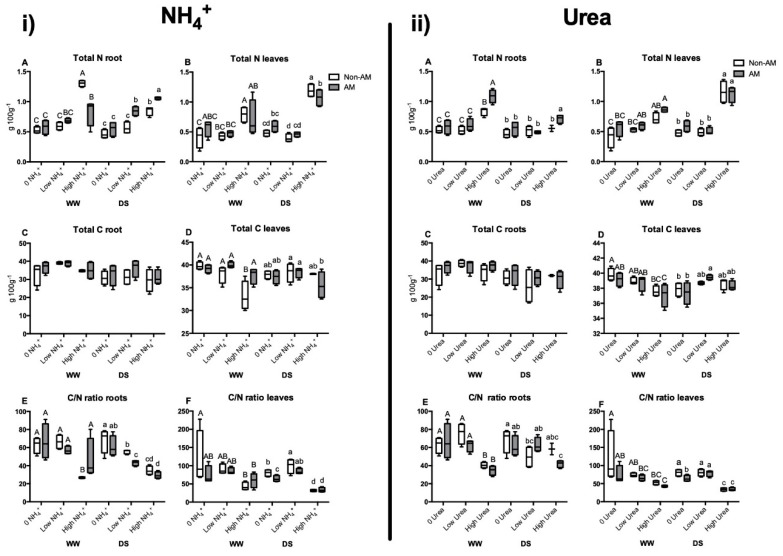
Total N ((**i**) **A**,**B**) for NH_4_^+^ experiment and **ii A** and **ii B** for the urea experiment) and C ((**i**) **C**) and ((**i**) **D**) for NH_4_^+^ experiment and ((**ii**) **C**) and ((**ii**) **D**) for the urea experiment) and ratio C/N ((**i**) **E**) and ((**i**) **F**) for NH_4_^+^ experiment and ((**ii**) **E**) and ((**ii**) **F**) for the urea experiment) in roots and leaves of maize plants inoculated or not with the AM fungus *Rhizophagus irregularis* (AM), submitted to two water regimes (well-watered, WW or drought stress, DS, for 14 days) and grown under different ammonium or urea concentrations (0 N added; low N, 3 µM or high N, 10 mM). Data represents the means of five biological replicates ± SE. Different letter (uppercase for WW treatments and lowercase for DS treatments) indicates significant differences between treatments (*p* < 0.05) based on Duncan’s test.

Under WW conditions, total nitrogen levels in roots and leaves were differently affected by N form and concentration. In NH_4_^+^-fertilized plants, high NH_4_^+^ application increased N content in roots, especially in non-AM plants. On the contrary, under DS conditions AM plants increased more total N concentration than non-AM plants (Figure 3i(A)). In leaves, the increase under WW conditions was similar in non-AM and AM plants when fertilized with high NH_4_^+^ concentration; but under DS the increase of N content was slightly higher in non-AM plants (Figure 3i(B)). In the case of urea, the total N in roots increased under high urea concentration, especially in AM plants under WW conditions and only in these plants under DS conditions (Figure 3ii(A)). Under DS conditions, total N concentration in leaves was significantly increased in both non-AM and AM plants fertilized with high urea level as compared to the other treatments (Figure 3ii(B)).

Total carbon (C) content in roots of plants fertilized with different N forms and concentrations remained constant in roots under the different experimental conditions (Figure 3i(C) and Figure 3ii(C)). In leaves, total C decreased in non-AM plants receiving high NH_4_^+^ supply under WW conditions and slightly decreased under DS conditions in AM plants with the same N treatment (Figure 3i(D)). Similarly, total C in leaves decreased with high urea fertilization under WW conditions. It was also low under drought stress in control plants non-fertilized with N, regardless of inoculation treatment (Figure 3ii(D)).

The C/N ratio in roots of NH_4_^+^-fertilized plants under WW conditions was reduced in non-AM plants receiving high N fertilization, while under DS, the ratio was progressively decreasing when increasing N fertilization level, both in AM and in non-AM plants (Figure 3i(E)). A similar pattern was observed in C/N ratio in roots of urea-treated plants (Figure 3ii(E)). In leaves under WW conditions, the ratio decreased also with high NH_4_^+^ application both in non-AM and AM plants. Under DS, the C/N ratio followed a similar trend with NH_4_^+^ and urea fertilization. Thus, it decreased in non-AM plants compared to AM plants when plants were not fertilized with N. The ratio increased in both inoculation treatments under low-N fertilization and it decreased in both inoculation treatments under high NH_4_^+^ fertilization (Figure 3i(F) and Figure 3ii(F)).

### 2.4. Accumulation of New Nitrogen in Tissues

The calculation of the percentage of new ^15^N nitrogen in the different analyzed tissues showed that the higher proportion of newly incorporated ^15^N was found in non-AM plants fertilized with low N concentration (independently of the N form and of the water regime). Thus, under well-watered conditions higher proportion of new ^15^N was found in leaves and stems of non-AM plants fertilized with low NH_4_^+^ (Table 2). Under drought stress, higher values were found in leaves, stems and roots of non-AM plants fertilized with low NH_4_^+^. In low urea-fertilized plants, a higher proportion of new ^15^N was found in leaves of non-AM plants under well-watered conditions and in leaves and stems of non-AM plants under drought stress conditions (Table 2).

### 2.5. Net Photosynthesis (A_N_) and Stomatal Conductance (gs)

**Figure 4:**
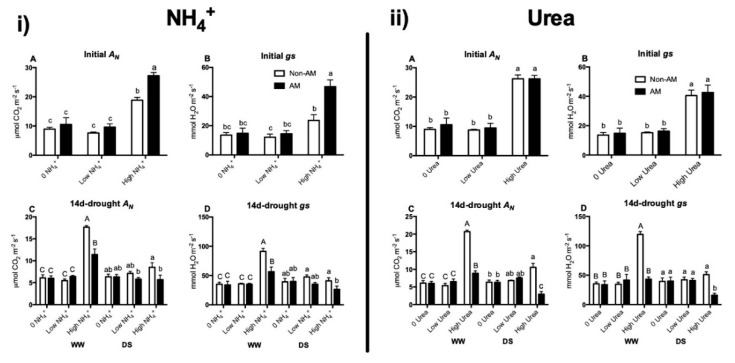
Net photosynthesis (*A_N_*) and stomatal conductance (*gs*) in maize plants inoculated or not with the AM fungus *Rhizophagus irregularis* (AM), submitted to two water regimes (well-watered, WW or drought stress, DS for 14 days) and grown under different urea or ammonium concentrations (0 N added; low N, 3 µM or high N, 10 mM). Parameters were measured before stress treatment (Initial values) ((**i**) **A**) and ((**i**) **B**) for NH_4_^+^ experiment and ((**ii**) **A**) and ((**ii**) **B**) for the urea experiment) or 14 days after starting the drought stress treatment (14d-drought) ((**i**) **C** and ((**i**) **D** for NH_4_^+^ experiment and ((**ii**) **C**) and ((**ii**) **D**) for the urea experiment). Data represents the means of five biological replicates ± SE. Different letter (uppercase for WW treatments and lowercase for DS treatments) indicates significant differences between treatments (*p* < 0.05) based on Duncan’s test.

Initial *A_N_* (before DS treatment) was not affected by low NH_4_^+^ or urea concentrations, and levels remained similar to those of plants unfertilized with N. However, under high N supply, *A_N_* significantly increased with both N forms. Interestingly, mycorrhization additionally increased net photosynthesis under high NH_4_^+^ supply compared to non-AM plants, while in urea-fertilized plants similar levels were observed in AM and non-AM plants (Figure 4i(A) and Figure 4ii(A)). The same pattern was observed for initial stomatal conductance (Figure 4i(B) and Figure 4ii(B)).

Fourteen days after drought stress imposition, *A_N_* and gs maintained similar trends of initial values in WW plants. Nevertheless, during DS, *A_N_* and gs values were generally decreased. In both ammonium- and urea-fertilized plants, a drop in *A_N_* and gs under high N fertilization in AM plants resulted in significant differences compared to non-AM plants (Figure 4i(C) and Figure 4i(D); Figure 4ii(C) and Figure 4ii(D)).

### 2.6. Root mRNA Levels of Maize Aquaporins

The mRNA relative abundances of eight maize aquaporins (*ZmPIP1;1*, *ZmPIP1;3*, *ZmPIP2;2*, *ZmPIP2;4*, *ZmTIP1;1*, *ZmTIP2;3*, *ZmTIP4;1* and *ZmNIP2;1*) that were selected in a previous study as regulated by the AM symbiosis [24] was analyzed. From these eight aquaporin genes, *ZmPIP1;1*, *ZmPIP1;3*, *ZmPIP2;2*, and *ZmTIP2;3* did not show significant differences among treatments, regardless of NH_4_^+^ or urea application. Thus, their results are not presented here.

**Figure 5:**
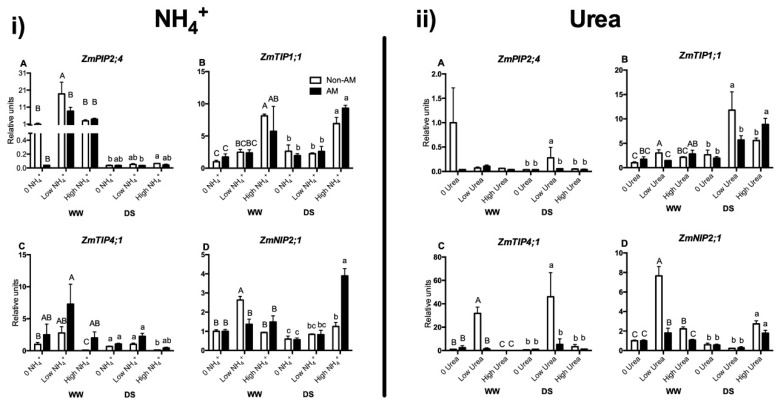
Relative root expression levels for *ZmPIP2;4* ((**i**) **A**) for NH_4_^+^ experiment and ((**ii**) **A**) for the urea experiment)*, ZmTIP1;1* ((**i**) **B**) for NH_4_^+^ experiment and ((**ii**) **B**) for the urea experiment)*, ZmTIP4;1* ((**i**) **C**) for NH_4_^+^ experiment and ((**ii**) **C**) for the urea experiment) and *ZmNIP2;2* ((**i**) **D**) for NH_4_^+^ experiment and ((**ii**) **D**) for the urea experiment) genes. Maize plants were inoculated or not with the AM fungus *Rhizophagus irregularis* (AM), submitted to two water regimes (well-watered, WW or drought stress, DS for 14 days) and grown under different ammonium or urea concentrations (0 N added; low N, 3 µM or high N, 10 mM). Data represents the means of three biological replicates ± SE. Different letter (uppercase for WW treatments and lowercase for DS treatments) indicates significant differences between treatments (*p* < 0.05) based on Duncan’s test.

*ZmPIP2;4* was up-regulated by low NH_4_^+^ level in non-AM plants under well-watered conditions (Figure 5i(A)). In the urea experiment, significant differences were only observed under drought stress conditions, where the gene was also up-regulated in non-AM plants by low urea application (Figure 5ii(A)).

*ZmTIP1;1* relative root transcript abundance increased under high NH_4_^+^ levels in both AM and non-AM plants under WW and DS conditions as compared to the other NH_4_^+^ concentrations (Figure 5i(B)). When plants were fertilized with urea, an increase in root transcript abundance was observed in non-AM plants receiving low urea fertilization under both well-watered and drought stress conditions, and in AM plants receiving high urea fertilization under drought stress conditions (Figure 5ii(B)).

In the case of *ZmTIP4;1* relative transcript abundance, in the NH_4_^+^ experiment the gene was up-regulated in AM plants under low N fertilization and WW conditions. In contrast, it was down-regulated in non-AM plants under high N level, regardless of the water regime (Figure 5i(C). In the experiment with urea a significant up-regulation *of ZmTIP4;1* occurred in non-AM plants with low urea concentration under both WW and DS conditions, as compared to the other treatments, being especially high under DS conditions (Figure 5ii(C).

*ZmNIP2;1* transcript levels increased in non-AM plants with low NH_4_^+^ under WW conditions, and also in AM plants with high NH_4_^+^ under DS conditions (Figure 5i(D). In analogy with this, the gene was also up-regulated under WW conditions in non-AM plants receiving low urea fertilization and to a lesser extent in AM plants. The gene was also up-regulated in non-AM plants with high urea under the same watering conditions, although the regulation was weaker in this case. Under DS, plants receiving high urea showed also an increase in *ZmNIP2;1* transcript levels compared to the other DS treatments, regardless of AM inoculation (Figure 5ii(D).

## 3. Discussion

Although many aquaporins are highly selective for water, the selectivity filters of plant aquaporins show a high divergence, suggesting wide functional diversity for these proteins [25]. Thus, it became increasingly clear that apart from water, urea, and ammonia, aquaporins can also transport other small neutral molecules such as non-electrolyte acetamide, long polyols, short polyols including glycerol, CO_2_, O_2_, purines and pyrimidines, silicic acid, boric, arsenic and germanic acids, lactic acid or hydrogen peroxide, and related oxy-radicals [26]. This fact highlights the paramount relevance of aquaporins for plant physiology. For instance, the ability of aquaporins to transport N compounds pointed to important roles for aquaporins in N mobilization and metabolism. The diffusion of CO_2_ through aquaporins suggests a role in carbon fixation. Silicon uptake and metabolism seem to be crucial for responses to biotic and abiotic stresses and boron is closely related with nutrition and structural development. The ability of aquaporins to transport H_2_O_2_ points to potential involvement in stress signaling and responses [22].

Under drought stress conditions, the uptake of soil nutrients by plant roots and subsequent mobilization to aerial parts is hampered by low soil water availability. The AM symbiosis was shown to alleviate limited soil nutrients availability, including N, under different environmental conditions [10,11] and to improve plant performance under both biotic and abiotic stresses [27]. In the case of drought, several mechanisms affecting plant physiology were proposed to explain the enhanced drought tolerance of AM plants [28,29]. However, given the fact that the AM symbiosis regulates a wide number of plant aquaporins under drought stress [22] and that besides water, some of these aquaporins can transport ammonium or urea, it is plausible that these AM-regulated aquaporins may be involved in the plant mobilization of N forms, contributing in this way to the enhanced drought tolerance of AM plants. This study aims to shed light on this hypothesis. Moreover, this study also aims to further understand how the AM symbiosis affects N balance in plants depending on N form and supply and how this combination of treatments affects plant physiology and performance under drought stress. The importance of plant-microbe interactions for reducing chemical fertilizers and inputs while maintaining normal development and plant growth is attracting more attention [30]. Furthermore, N fertilization strongly affects crop productivity, and the increase in plant N-uptake efficiency and its mobilization capacity could reduce the consumption of N fertilizers.

### 3.1. Physiological Effects of AM Symbiosis under Different N Supply and Watering Conditions

As reported in numerous studies with arbuscular mycorrhizal symbioses and in previous experiments with similar conditions [31,32], the total biomass (represented by plant FW) was generally enhanced by the AM symbiosis under both watering conditions. This effect, however, was not observed under high N supply (regardless of the N form, Table 1). This is understandable, as when N is in excess in the medium, the plant can directly take it, and AM symbiosis does not suppose an additional benefit for nutrient acquisition. Consistent with this, a recent study also found that the growth-promoting effect of the AM fungus was only observed under low N conditions [33]. High nitrogen supply increased leaf N content, especially during drought stress, and it was positively correlated with plant growth (Figure 3i(B) and Figure 3ii(B)). AM plants did not show higher N levels in leaves. However, AM plants increased N levels in roots compared to non-AM plants in urea-fed plants under either WW or DS and under DS in plants irrigated with high NH_4_^+^ (Figure 3i(A) and Figure 3ii(A)).

Low MDA levels, produced as the result of lipid peroxidation are usually related to higher membrane stability [34]. Our study showed that without N supply under WW conditions, the levels of MDA were higher compared to the other treatments, regardless of the N form (Table 1). This fact suggests that nutrient status affects cell membrane stability. Under drought stress conditions, the AM fungus generally decreased MDA levels regardless of N concentration or form, which is in accordance with previous studies [24,35].

SPAD values estimate chlorophyll content of the leaves and can be correlated with N concentration per leaf area, although this relationship is very variable depending on environmental factors and plant species [36]. Moreover, leaf chlorophyll content is a determinant factor in plant photosynthesis [37]. In our study, SPAD values were enhanced with the increase in N concentration with both urea and NH_4_^+^ and in both well-watered and drought stressed plants. In some cases, when the concentration of N was low, mycorrhization was found to increase SPAD values, but once more, under high N supply, the symbiosis did not enhance this parameter (Table 1).

Altogether, physiological data showed that maize plants grew well under the highest N concentration and grew with restriction under the two low N levels. This may be related to the plant species, or variety used [38].

Under well-watered conditions, *A_N_* and *gs* were enhanced by high N concentrations (regardless of N form) at the two measured time points (Figure 4). This effect was also shown in other studies [39,40]. During water deprivation, stomatal closure and increase in the resistance of CO_2_ diffusion commonly lead to a decrease in photosynthetic rate [41]. In our study, drought stress negatively impacted *A_N_* and *gs* under both high NH_4_^+^ and urea supply, especially in AM plants after 14 days of drought (Figure 4). In fact, after 14 days of drought, AM plants fertilized with high NH_4_^+^ or urea doses had even lower *A_N_* and *gs* levels than non-AM plants. As explained above, when plant growth is not limited by soil N availability, mycorrhizal inoculation might not suppose an advantage and the plant may reduce its dependence on the AM fungus.

### 3.2. N Acquisition by the Plant

In this study, the use of ^15^N-labelled urea and ammonium showed that non-AM plants receiving low N supply enhanced most the uptake of new N under both well-watered or drought stress conditions, being reflected by the measured percentage of ^15^N in leaves, stems and roots. This may be due to a higher N demand in plants fertilized with low N levels, as comparted to those receiving high N levels or to AM plants. Indeed, it is likely that the high nutrient concentration under high urea or ammonium supply inhibited the uptake of new N. The effect was observed with both ammonium and urea, highlighting that the two nutrients are very similar in their assimilation pathways, as previously suggested [4]. However, with urea nutrition, these differences were only observed in leaves or in stem in the case of drought stressed plants. In the case of AM plants, the transport of labelled ^15^N was generally low, which may reflect a lower N demand in these plants during the short time of ^15^N labelling (3h) due to a general better N nutrition in AM plants. In other study, the transport of ^15^N-NH_4_^+^ to the AM plant was lower compared to NO_3_^−^ nutrition in wheat [42].

### 3.3. AQP Relative Gene Expression Levels Are Affected by N Supply and Form

Generally, the patterns of root AQPs gene expression were different with the application of NH_4_^+^ or urea and also differed in response to N levels in our experiment (Figure 5). This fact suggests that the plant discriminated between the two N forms and that at least not all the applied urea was transformed into NH_4_^+^ in the soil. N acquisition is commonly affected by water flow from the soil and through the plants. In this sense, aquaporins were related to N balance in several independent studies, and their overexpression positively affected total N uptake and transpiration rate [21].

Two main families of AQPs are potentially involved in the mobilization of N: tonoplast intrinsic proteins (TIPs) and nodulin 26-like intrinsic proteins (NIPs) [41]. This may explain why *ZmPIP1;1*, *ZmPIP1;3*, *ZmPIP2;2* and also *ZmTIP2;3* did not show significant differences among treatments, regardless of NH_4_^+^ or urea application. In contrast, ZmTIP1;1 and ZmNIP2;1 proteins were shown to transport urea when expressed in yeast. In addition, ZmTIP1;1 was found to transport NH_4_^+^ in the same heterologous system in previous studies [22,23]. Our results indicate that ZmTIP1;1 could be transporting in planta both urea and ammonium, as the gene was up-regulated under high NH_4_^+^ concentrations (especially under DS conditions) and under low or high urea concentrations under WW or DS conditions (Figure 5i(B) and Figure 5ii(B)). The induction of *ZmTIP1;1* by the AM symbiosis under high N conditions may be related to the storage in the vacuole of excess N taken up by the plants under such conditions of abundant N, as this aquaporins is from tonoplast and could be involved in cellular N homeostasis [43]. In the case of ZmNIP2;1, our data suggests that together with urea it could be transporting NH_4_^+^, as the gene was up-regulated with low NH_4_^+^ supply under WW and with high NH_4_^+^ supply under DS (Figure 5i(D)). The differential regulation of *ZmTIP4;1* and *ZmPIP2;4* with urea fertilization and *ZmPIP2;4* with NH_4_^+^ supply made us hypothesize that these two AQPs may also play a role in N homeostasis in planta. Besides, the genes found to be regulated by N status were at the same time differentially regulated by the AM symbiosis. For instance, *ZmNIP2;1* was strongly up-regulated in AM plants with high NH_4_^+^ under DS but not in non-AM plants, and in non-AM plants with low NH_4_^+^ but not in AM plants (Figure 5i(D)). On the contrary, *ZmTIP4;1* was up-regulated by low urea supply under both water treatments in non-AM plants but unaffected in AM plants (Figure 5ii(C)). These results support a differential regulation of some plant aquaporins when the plant is colonized by the AM fungus.

## 4. Materials and Methods

### 4.1. Experimental Design

The experiment consisted of a randomized block design with (1) two inoculation treatments: non-inoculated plants (Non-AM) and plants inoculated with an arbuscular mycorrhizal fungus (AM plants); (2) two water regimes: WW plants and DS plants; and (3) two sources of nitrogen: urea and ammonium applied at three different concentrations: No N added, low concentration (3 µM) and high concentration (10 mM). The experiment consisted of six replicates per treatment and plants receiving no N fertilization were common for the urea and ammonium experiments, resulting in a total of 120 plants.

### 4.2. Soil and Biological Materials

The growing substrate consisted of a mixture of soil and sand (1:9 v/v). The soil (collected at the grounds of IFAPA, Granada, Spain) was sieved (2 mm), diluted with quartz-sand (<1 mm) and sterilized by steaming (100 °C for 1 h) on three consecutive days. It had a pH of 8.1 (water); 0.85% organic matter, nutrient concentrations (mg kg^−1^): P, 10 (NaHCO_3_-extractable P); N, 1; K, 110. The soil texture was made of 47.1% silt, 38.3% sand and 14.6% clay.

Seeds of *Zea mays* L. cultivar PR34B39 were provided by Pioneer Hi-Bred (DuPont, Sevilla, Spain), and were pre-germinated in sand for five days and then transferred to 1.5 L pots containing 1250 g of the above described substrate. At planting time, half of the plants were inoculated with ten grams of AM inoculum with *Rhizophagus irregularis* (Schenck and Smith), strain EEZ 58. The inoculum consisted of soil, spores, mycelia, and infected root fragments. Non-inoculated plants received a 10 mL aliquot of an inoculum filtrate (<20 µm), in order to provide the microbial population accompanying the AM inoculum but free of AM propagules.

### 4.3. Growing Conditions

Plants were cultivated for eight weeks under greenhouse conditions (photosynthetic photon flux density of ca. 800 µmol m^−2^ s^−1^, 25/20 ℃, 16/8 light dark period and 50–60% RH). Each plant received 50 mL of Hoagland nutrient solution [44] three times per week. The solution was modified to contain only 25% of P, in order to avoid inhibition of mycorrhization. The Hoagland solution was also modified to provide the different nitrogen forms and concentrations as explained above. Ammonium was applied in the nutrient solution as (NH_4_)_2_S_2_O_8_. On alternate days plants received the same amount of water. The drought stress treatment was imposed the last two weeks to half of the plants by irrigating with half of the water/Hoagland volume of well-watered plants (25 mL vs. 50 mL). To avoid nutrient deficiency, droughted treatments received 2X Hoagland nutrient solution. The water stress imposed was similar to previous studies and considered to be a severe stress [24,31,32,45].

A pulse with labelled 10% ^15^N was applied with the nutrient solution three hours before harvest to four plants per treatment in two different forms: Urea-^15^N_2_ (98 atom % ^15^N), and ammonium-^15^N_2_ sulphate (60 atom % ^15^N), Sigma Aldrich.

### 4.4. Parameters Measured

#### 4.4.1. Biomass Production and Symbiotic Development

The roots and aerial parts of six plants per treatment were used for fresh weight measurement at harvest (8 weeks after sowing).

Aliquots of three maize roots per treatment were used for staining with Trypan blue following the procedure described by Phillips and Hayman [46], in order to differentiate AM fungal structures. The gridline intersect method [47] was used in those roots to calculate the extent of mycorrhizal colonization.

#### 4.4.2. Oxidative Damage to Lipids

Lipid peroxides were extracted from three fresh leaves per treatment by grinding 500 mg of tissue with 6 mL of 100 mM potassium phosphate buffer (pH 7) and quantified as described in Quiroga et al. [24]. Lipid peroxidation was estimated as the content of 2-thiobarbituric acid-reactive substances (TBARS) and expressed as equivalents of malondialdehyde (MDA) according to Halliwell and Gutteridge [48].

#### 4.4.3. Leaf Chlorophyll Content

Chlorophyll content was estimated in the second fully expanded youngest leaf of six plants per treatment four hours after the onset of photoperiod and one day before harvest by using a Chlorophyll Content Measurement System CL-01 (SPAD, Hansatech Instruments Ltd., Norfolk, UK). This instrument determines relative chlorophyll content in leaf samples by measuring dual optical absorbances (620 and 940 nm wavelengths).

#### 4.4.4. Mineral Analysis

Mineral analysis was performed in the INAN-EEZ Institute in Armilla, Granada, Spain. The equipment used was an elemental analyzer Leco TruSpec CN, which detects Nitrogen in the sample by Dumas method, including a complete combustion of the sample at 950 ℃ with high pressure oxygen, chemical reduction of nitrogen oxides to molecular nitrogen and analysis of this molecular nitrogen with a thermal conductivity detector (TCD). Carbon was detected as carbon dioxide (from the sample combustion) by an infrared detector. Quantification was performed with certified standards from Leco.

#### 4.4.5. Net Photosynthesis and Stomatal Conductance

Stomatal conductance (gs) and net photosynthesis (A*_N_*) were measured in the second fully expanded youngest leaf of five plants per treatment at three time points: one week before starting the drought stress treatment (6 weeks of growing), after 7 days of drought stress and after 14 days of drought treatment. Both parameters were measured by using a portable photosystem system LI-6400 (LICOR Biosciences, Lincoln, NE, USA) two hours after sunrise. Measurements were performed at an ambient CO_2_ concentration of 390 µmol mol^−1^, temperature of 25/30 ^0^C, 50 ± 5% relative humidity and a photosynthetic photon flux density (PPFD) of 1000 µmol m^−2^s^−1^.

#### 4.4.6. RT-qPCR

Total RNA of three biological replicates from maize roots was extracted as described in Quiroga et al. [24]. Maxima H Minus first strand cDNA kit (Thermo Scientific ^TM^) was used for the synthesis of first strand cDNA by using 1 µg of purified total RNA, according to the manufacturer’s instructions.

The mRNA relative abundances of eight maize aquaporins (*ZmPIP1;1*, *ZmPIP1;3*, *ZmPIP2;2*, *ZmPIP2;4*, *ZmTIP1;1*, *ZmTIP2;3*, *ZmTIP4;1* and *ZmNIP2;1*) were measured by real time q-RT-PCR using 1 µL of diluted cDNA (1:9) and PowerUp^TM^ SYBR^TM^ Green Master Mix in a QuantStudio^TM^ 3 system (Thermo Fisher Scientific). Annealing temperature was 58ºC for all primers and the reaction was repeated for 40 cycles. Four reference genes were tested for normalization of gene expression values: elongation factor 1 (gi:2282583), poliubiquitin (gi:248338), tubulin (gi:450292) and GAPDH (gi:22237) [22]. “NormFinder” algorithm [49] (https://moma.dk/normfinder-software) was used to select the best-performing reference gene under our experimental conditions. Thus, elongation factor 1 (gi:2282583) was used for normalization. The relative abundance of transcripts was calculated using the 2^-ΔΔCt^ method [50]. Three biological replicates were used per treatment and the threshold cycle (Ct) of each biological sample was determined in duplicate. Negative controls without cDNA were used in all PCR reactions.

#### 4.4.7. Estimation of ^15^N Isotopic Abundances

Sample analysis for ^15^N composition was carried out at the Stable Isotope Laboratory of the Instituto Andaluz de Ciencias de la Tierra in Granada (CSIC-UGR), Spain. Stable isotope (δ^15^N) analysis of leaf, stem and root samples were performed on a Carlo Elba NC1500 (Milan, Italy) elemental analyzer coupled on-line via a ConFlow III with a Delta Plus XP (Thermo-Finnigan, Bremen, Germany) mass spectrometer (EA-IRMS). Commercial N_2_ was used as the internal standard for the N isotopic analyses.

### 4.5. Statistical Analysis

The SPSS Statistics (Version 26, IBM Analytics) was used to perform data analysis. The normality and homogeneity of variances were tested. Some parameters were logarithmically transformed to achieve homogeneity of variance. Data obtained for ammonium and urea were analyzed separately by using one-way ANOVA in order to facilitate interpretation of results in this experiment that contains up to four factors. Duncan’s multiple range test was used to pairwise multiple comparisons between treatments at α = 0.05.

## 5. Conclusions

AM symbiosis improved plant biomass in absence of N fertilization or under low urea or ammonium fertilization, regardless of the watering regime. In contrast, under high N supply, no effect of AM symbiosis on plant development was observed. This effect was associated with reduced oxidative damage to lipids and increased N accumulation in plant tissues. High N fertilization with either ammonium or urea enhanced net photosynthesis (*A_N_*) and stomatal conductance (*gs*) values in plants maintained under well-watered conditions, but 14 days after drought stress imposition these parameters declined in AM plants fertilized with high N doses. The aquaporin ZmTIP1;1 could be transporting in planta both urea and ammonium, as the gene was up-regulated under high NH_4_^+^ concentrations and under low and high urea concentrations. The differential regulation of *ZmTIP4;1* and *ZmPIP2;4* with urea fertilization and of *ZmPIP2;4* with NH_4_^+^ supply suggests that these two aquaporins may also play a role in N mobilization in planta. At the same time, the aquaporin genes found to be regulated by N status were also differentially regulated by the AM symbiosis, suggesting a possible role of these aquaporins in the AM-mediated plant N homeostasis that deserves future studies.

## Figures and Tables

**Table 1:** Plant fresh weight (FW), percentage of mycorrhizal root length, oxidative damage to lipids (as malondialdehyde MDA, equivalents) and SPAD values in maize plants inoculated or not with the AM fungus *Rhizophagus irregularis* (AM), submitted to two water regimes (well-watered, WW or drought stress, DS for 14 days) and grown under different ammonium or urea concentrations (0 N added; low N, 3 µM or high N, 10 mM).

			Plant FW (g plant^−1^)		Mycorrhization (%)		MDA (nmol g FW^−1^)		SPAD Values	
			NH_4_^+^	Urea	NH_4_^+^	Urea	NH_4_^+^	Urea	NH_4_^+^	Urea
WW	0 N	Non-AM	20.4 ± 0.52D*	20.4 ± 0.52C*	n.d	n.d	37.7 ± 3.58A*	37.7 ± 3.58A*	1.6 ± 0.11CD*	1.6 ± 0.11D*
		AM	28.0 ± 1.12C*	28.0 ± 1.12B*	45.1 ± 1.74AB*	45.1 ± 1.74AB*	35.7 ± 3.37A*	35.7 ± 3.37A*	1.9 ± 0.06B*	1.9 ± 0.06C*
	Low N	Non-AM	13.8 ± 0.94E	13.6 ± 0.51D	n.d.	n.d.	34.4 ± 3.70A	14.4 ± 3.09B	1.6 ± 0.07D	1.8 ± 0.08CD
		AM	25.4 ± 0.51C	29.3 ± 0.65B	52.5 ± 2.33AB	43.1 ± 2.27B	8.74 ± 3.39B	14.3 ± 2.95B	1.9 ± 0.17BC	1.8 ± 0.13CD
	High N	Non-AM	124.27 ± 0.94A	119.2 ± 2.24A	n.d.	n.d.	6.34 ± 2.17B	9.87 ± 0.50B	5.6 ± 0.24A	6.5 ± 0.13A
		AM	107.9 ± 3.81B	120.9 ± 2.79A	46.1 ± 14.4AB	64.9 ± 1.52A	9.56 ± 2.94B	11.4 ± 2.17B	4.9 ± 0.19A	4.7 ± 0.14B
DS	0 N	Non-AM	18.6 ± 0.71c*	18.6 ± 0.71d*	n.d	n.d	43.7 ± 2.20a*	43.7 ± 2.20a*	1.7 ± 0.09bc*	1.7 ± 0.09c*
		AM	22.87 ± 0.34b*	22.87 ± 0.34c*	39.1 ± 2.93b*	39.1 ± 2.93b*	30.1 ± 2.26b*	30.1 ± 2.26b*	1.8 ± 0.09b*	1.8 ± 0.09bc*
	Low N	Non-AM	12.56 ± 0.19d	13.7 ± 0.65e	n.d.	n.d.	50.9 ± 5.30a	31.5 ± 4.15b	1.7 ± 0.12bc	1.7 ± 0.10c
		AM	19.1 ± 2.50c	27.4 ± 0.80b	37.3 ± 3.41b	53.55 ± 10.7ab	28.4 ± 4.93b	18.8 ± 2.35cd	1.5 ± 0.09c	2.1 ± 0.19b
	High N	Non-AM	91.43 ± 2.87a	89.6 ± 2.14a	n.d.	n.d.	12.4 ± 1.30b	24.9 ± 3.91bc	5.3 ± 0.21a	5.6 ± 0.20a
		AM	85.5 ± 1.03a	85.1 ± 0.71a	38.7 ± 1.66b	43.9 ± 7.38ab	14.0 ± 2.34b	11.3 ± 2.37d	5.6 ± 0.44a	6.1 ± 0.43a

Data represents the means of six biological replicates ± SE for plant dry weight, three biological replicates ± SE for mycorrhization and MDA and six biological replicates for SPAD. Different letter (uppercase for WW treatments and lowercase for DS treatments) indicates significant differences between treatments (*p* < 0.05) based on Duncan’s test. Asterisks indicate that the plants cultivated with 0 N are common to the NH_4_^+^ and the urea experiments. n.d. means non-detected.

**Table 2:** Atoms % ^15^N incorporated in leaf, stem and root of maize plants inoculated or not with the AM fungus *Rhizophagus irregularis* (AM), submitted to two water regimes (well-watered, WW or drought stress, DS for 14 days) and fertilized with low (3 µM) or high (10 mM) ammonium or urea concentrations during the growing period. Plants were labelled with ammonium sulphate (60 atom % ^15^N) or urea (98 atom % ^15^N) for 3h before harvest.

			At % ^15^N					
			NH_4_^+^			Urea		
			Leaf	Stem	Root	Leaf	Stem	Root
WW	Low N	Non-AM	1.31 ± 0.17 A	1.76 ± 0.23 A	0.46 ± 0.15 AB	0.73 ± 0.05 A	0.87 ± 0.18 A	0.33 ± 0.04 A
		AM	0.37 ± 0.10 B	0.73 ± 0.09 B	0.58 ± 0.21 A	0.25 ± 0.04 C	0.57 ± 0.15 A	0.57 ± 0.17 A
	High N	Non-AM	0.41 ± 0.06 B	0.90 ± 0.06 B	0.16 ± 0.06 B	0.33 ± 0.06 BC	0.89 ± 0.06 A	0.50 ± 0.04 A
		AM	0.50 ± 0.07 B	0.94 ± 0.04 B	0.58 ± 0.11 A	0.40 ± 0.03 B	0.79 ± 0.02 A	0.45 ± 0.05 A
DS	Low N	Non-AM	1.23 ± 0.07 a	1.03 ± 0.08 a	0.80 ± 0.07 a	1.70 ± 0.24 a	1.61 ± 0.25 a	0.42 ± 0.13 a
		AM	0.34 ± 0.07 b	0.52 ± 0.18 b	0.62 ± 0.23 ab	0.42 ± 0.05 b	0.66 ± 0.11 b	0.35 ± 0.04 a
	High N	Non-AM	0.19 ± 0.07 b	0.35 ± 0.07 b	0.36 ± 0.12 b	0.16 ± 0.05 b	0.29 ± 0.08 b	0.36 ± 0.07 a
		AM	0.34 ± 0.02 b	0.45 ± 0.07 b	0.30 ± 0.07 b	0.18 ± 0.03 b	0.52 ± 0.02 b	0.53 ± 0.04 a

Data represents the means of four biological replicates ± SE. Different letter (uppercase for WW treatments and lowercase for DS treatments) indicates significant differences between treatments (*p* < 0.05) based on Duncan’s test

## Supplementary Materials

The following are available online at https://www.mdpi.com/2223-7747/9/2/148/s1. Table S1: one-way ANOVA for data presented in this study. Table S2: multifactorial ANOVA for data presented in this study.
