# Supplementary material for: Elucidating the Possible Involvement of Maize Aquaporins and Arbuscular Mycorrhizal Symbiosis in the Plant Ammonium and Urea Transport under Drought Stress Conditions
Source: Plants (Basel). 2020 Jan 23;9(2):148. doi: 10.3390/plants9020148 (PMC7076390; doi:10.3390/plants9020148)
Supplement: Supplementary file 1 [file plants-09-00148-s001.zip › supplementary/Table S1 One-way ANOVA.docx]

|  |  | **NH_4_^+^** | | | | | | | | | |
| --- | --- | --- | --- | --- | --- | --- | --- | --- | --- | --- | --- |
|  |  | **WW** | | | | | **DS** | | | | |
|  |  | **Sum of Squares** | **df** | **Mean Square** | **F** | **Sig.** | **Sum of Squares** | **df** | **Mean Square** | **F** | **Sig.** |
| **Plant FW** | **Between Groups** | 4.721 | 5 | 0.944 | 652.52 | 0.000 | 4.135 | 5 | 0.827 | 156.8 | 0 |
|  | **Within Groups** | 0.043 | 30 | 0.001 |  |  | 0.158 | 30 | 0.005 |  |  |
|  | **Total** | 4.764 | 35 |  |  |  | 4.294 | 35 |  |  |  |
| **Mycorrhization** | **Between Groups** | 95.639 | 2 | 47.82 | 0.221 | 0.808 | 68.135 | 2 | 34.067 | 0.495 | 0.63 |
|  | **Within Groups** | 1299.943 | 6 | 216.657 |  |  | 413.344 | 6 | 68.891 |  |  |
|  | **Total** | 1395.582 | 8 |  |  |  | 481.479 | 8 |  |  |  |
| **MDA** | **Between Groups** | 2.213 | 5 | 0.443 | 13.049 | 0.000 | 2.067 | 5 | 0.413 | 16.752 | 0 |
|  | **Within Groups** | 0.407 | 12 | 0.034 |  |  | 0.296 | 12 | 0.025 |  |  |
|  | **Total** | 2.62 | 17 |  |  |  | 2.363 | 17 |  |  |  |
| **SPAD** | **Between Groups** | 1.955 | 5 | 0.391 | 104.62 | 0.000 | 2.211 | 5 | 0.442 | 97.635 | 0 |
|  | **Within Groups** | 0.112 | 30 | 0.004 |  |  | 0.136 | 30 | 0.005 |  |  |
|  | **Total** | 2.067 | 35 |  |  |  | 2.347 | 35 |  |  |  |

**One-way ANOVA for data shown in Table 1.**

|  |  | **Urea** | | | | | | | | | |
| --- | --- | --- | --- | --- | --- | --- | --- | --- | --- | --- | --- |
|  |  | **WW** | | | | | **DS** | | | | |
|  |  | **Sum of Squares** | **df** | **Mean Square** | **F** | **Sig.** | **Sum of Squares** | **df** | **Mean Square** | **F** | **Sig.** |
| **Plant FW** | **Between Groups** | 4.822 | 5 | 0.964 | 1113.385 | 0 | 3.565 | 5 | 0.713 | 690.767 | 0 |
|  | **Within Groups** | 0.026 | 30 | 0.001 |  |  | 0.031 | 30 | 0.001 |  |  |
|  | **Total** | 4.848 | 35 |  |  |  | 3.596 | 35 |  |  |  |
| **Mycorrhization** | **Between Groups** | 874.414 | 2 | 437.207 | 41.813 | 0 | 326.606 | 2 | 163.303 | 0.92 | 0.448 |
|  | **Within Groups** | 62.738 | 6 | 10.456 |  |  | 1065.364 | 6 | 177.561 |  |  |
|  | **Total** | 937.151 | 8 |  |  |  | 1391.971 | 8 |  |  |  |
| **MDA** | **Between Groups** | 2401.071 | 5 | 480.214 | 20.275 | 0 | 1875.728 | 5 | 375.146 | 13.965 | 0 |
|  | **Within Groups** | 284.214 | 12 | 23.685 |  |  | 322.351 | 12 | 26.863 |  |  |
|  | **Total** | 2685.286 | 17 |  |  |  | 2198.08 | 17 |  |  |  |
| **SPAD** | **Between Groups** | 2.078 | 5 | 0.416 | 150.728 | 0 | 2.172 | 5 | 0.434 | 95.407 | 0 |
|  | **Within Groups** | 0.083 | 30 | 0.003 |  |  | 0.137 | 30 | 0.005 |  |  |
|  | **Total** | 2.161 | 35 |  |  |  | 2.308 | 35 |  |  |  |

|  |  | **NH_4_^+^** | | | | | | | | | |
| --- | --- | --- | --- | --- | --- | --- | --- | --- | --- | --- | --- |
|  |  | **WW** | | | | | **DS** | | | | |
|  |  | **Sum of Squares** | **df** | **Mean Square** | **F** | **Sig.** | **Sum of Squares** | **df** | **Mean Square** | **F** | **Sig.** |
| **New N_Leaf** | **Between Groups** | 0.026 | 3 | 0.009 | 22.525 | 0.000 | 0.029 | 3 | 0.01 | 84.108 | 0 |
|  | **Within Groups** | 0.005 | 12 | 0 |  |  | 0.001 | 12 | 0 |  |  |
|  | **Total** | 0.03 | 15 |  |  |  | 0.031 | 15 |  |  |  |
| **New N_Stem** | **Between Groups** | 0.005 | 3 | 0.002 | 2.564 | 0.104 | 0.007 | 3 | 0.002 | 3.721 | 0.04 |
|  | **Within Groups** | 0.008 | 12 | 0.001 |  |  | 0.008 | 12 | 0.001 |  |  |
|  | **Total** | 0.013 | 15 |  |  |  | 0.015 | 15 |  |  |  |
| **New N_Root** | **Between Groups** | 0.027 | 3 | 0.009 | 17.601 | 0.000 | 0.012 | 3 | 0.004 | 9.878 | 0 |
|  | **Within Groups** | 0.006 | 12 | 0.001 |  |  | 0.005 | 12 | 0 |  |  |
|  | **Total** | 0.034 | 15 |  |  |  | 0.017 | 15 |  |  |  |

**One-way ANOVA for data shown in Table 2.**

|  |  | **Urea** | | | | | | | | | |
| --- | --- | --- | --- | --- | --- | --- | --- | --- | --- | --- | --- |
|  |  | **WW** | | | | | **DS** | | | | |
|  |  | **Sum of Squares** | **df** | **Mean Square** | **F** | **Sig.** | **Sum of Squares** | **df** | **Mean Square** | **F** | **Sig.** |
| **New N_Leaf** | **Between Groups** | 0.006 | 3 | 0.002 | 31.526 | 0 | 0.069 | 3 | 0.023 | 45.596 | 0 |
|  | **Within Groups** | 0.001 | 12 | 0 |  |  | 0.006 | 12 | 0.001 |  |  |
|  | **Total** | 0.007 | 15 |  |  |  | 0.075 | 15 |  |  |  |
| **New N_Stem** | **Between Groups** | 0.003 | 3 | 0.001 | 1.889 | 0.185 | 0.043 | 3 | 0.014 | 21.9 | 0 |
|  | **Within Groups** | 0.006 | 12 | 0 |  |  | 0.008 | 12 | 0.001 |  |  |
|  | **Total** | 0.008 | 15 |  |  |  | 0.051 | 15 |  |  |  |
| **New N_Root** | **Between Groups** | 0.001 | 3 | 0 | 1.706 | 0.219 | 0.001 | 3 | 0 | 1.524 | 0.259 |
|  | **Within Groups** | 0.003 | 12 | 0 |  |  | 0.002 | 12 | 0 |  |  |
|  | **Total** | 0.005 | 15 |  |  |  | 0.003 | 15 |  |  |  |

|  |  | **NH_4_^+^** | | | | | | | | | |
| --- | --- | --- | --- | --- | --- | --- | --- | --- | --- | --- | --- |
|  |  | **WW** | | | | | **DS** | | | | |
|  |  | **Sum of Squares** | **df** | **Mean Square** | **F** | **Sig.** | **Sum of Squares** | **df** | **Mean Square** | **F** | **Sig.** |
| **N total Root** | **Between Groups** | 1.678 | 5 | 0.336 | 27.016 | 0.000 | 1.067 | 5 | 0.213 | 30.973 | 0.000 |
|  | **Within Groups** | 0.224 | 18 | 0.012 |  |  | 0.124 | 18 | 0.007 |  |  |
|  | **Total** | 1.902 | 23 |  |  |  | 1.191 | 23 |  |  |  |
| **N total Leaves** | **Between Groups** | 0.289 | 5 | 0.058 | 3.255 | 0.029 | 0.592 | 5 | 0.118 | 16.042 | 0.000 |
|  | **Within Groups** | 0.319 | 18 | 0.018 |  |  | 0.133 | 18 | 0.007 |  |  |
|  | **Total** | 0.608 | 23 |  |  |  | 0.725 | 23 |  |  |  |
| **C total Root** | **Between Groups** | 114.76 | 5 | 22.952 | 1.853 | 0.153 | 116.107 | 5 | 23.221 | 0.938 | 0.480 |
|  | **Within Groups** | 222.981 | 18 | 12.388 |  |  | 445.459 | 18 | 24.748 |  |  |
|  | **Total** | 337.741 | 23 |  |  |  | 561.566 | 23 |  |  |  |
| **C total Leaves** | **Between Groups** | 0.019 | 5 | 0.004 | 7.912 | 0.000 | 0.003 | 5 | 0.001 | 1.688 | 0.189 |
|  | **Within Groups** | 0.009 | 18 | 0 |  |  | 0.007 | 18 | 0 |  |  |
|  | **Total** | 0.028 | 23 |  |  |  | 0.011 | 23 |  |  |  |
| **C/N Roots** | **Between Groups** | 4788.518 | 5 | 957.704 | 5.467 | 0.003 | 4727.871 | 5 | 945.574 | 15.071 | 0.000 |
|  | **Within Groups** | 3153.437 | 18 | 175.191 |  |  | 1129.377 | 18 | 62.743 |  |  |
|  | **Total** | 7941.955 | 23 |  |  |  | 5857.248 | 23 |  |  |  |
| **C/N Leaves** | **Between Groups** | 13797.16 | 5 | 2759.43 | 2.404 | 0.078 | 15422.11 | 5 | 3084.423 | 27.675 | 0.000 |
|  | **Within Groups** | 20661.82 | 18 | 1147.88 |  |  | 2006.147 | 18 | 111.453 |  |  |
|  | **Total** | 34458.98 | 23 |  |  |  | 17428.26 | 23 |  |  |  |

**One-way ANOVA for data shown in Figure 3.**

|  |  | **Urea** | | | | | | | | | |
| --- | --- | --- | --- | --- | --- | --- | --- | --- | --- | --- | --- |
|  |  | **WW** | | | | | **DS** | | | | |
|  |  | **Sum of Squares** | **df** | **Mean Square** | **F** | **Sig.** | **Sum of Squares** | **df** | **Mean Square** | **F** | **Sig.** |
| **N total Root** | **Between Groups** | 0.335 | 5 | 0.067 | 18.286 | 0.000 | 0.087 | 5 | 0.017 | 3.99 | 0.015 |
|  | **Within Groups** | 0.066 | 18 | 0.004 |  |  | 0.07 | 16 | 0.004 |  |  |
|  | **Total** | 0.401 | 23 |  |  |  | 0.157 | 21 |  |  |  |
| **N total Leaves** | **Between Groups** | 0.3 | 5 | 0.06 | 4.977 | 0.005 | 0.662 | 5 | 0.132 | 40.083 | 0.000 |
|  | **Within Groups** | 0.217 | 18 | 0.012 |  |  | 0.059 | 18 | 0.003 |  |  |
|  | **Total** | 0.517 | 23 |  |  |  | 0.722 | 23 |  |  |  |
| **C total Root** | **Between Groups** | 91.425 | 5 | 18.285 | 1.11 | 0.390 | 106.899 | 5 | 21.38 | 0.639 | 0.674 |
|  | **Within Groups** | 296.629 | 18 | 16.479 |  |  | 535.669 | 16 | 33.479 |  |  |
|  | **Total** | 388.054 | 23 |  |  |  | 642.568 | 21 |  |  |  |
| **C total Leaves** | **Between Groups** | 0.003 | 5 | 0.001 | 4.139 | 0.011 | 0.001 | 5 | 0 | 2.888 | 0.044 |
|  | **Within Groups** | 0.002 | 18 | 0 |  |  | 0.002 | 18 | 0 |  |  |
|  | **Total** | 0.005 | 23 |  |  |  | 0.003 | 23 |  |  |  |
| **C/N Roots** | **Between Groups** | 5063.393 | 5 | 1012.679 | 9.026 | 0.000 | 1807.432 | 5 | 361.486 | 3.432 | 0.027 |
|  | **Within Groups** | 2019.567 | 18 | 112.198 |  |  | 1685.086 | 16 | 105.318 |  |  |
|  | **Total** | 7082.959 | 23 |  |  |  | 3492.518 | 21 |  |  |  |
| **C/N Leaves** | **Between Groups** | 0.356 | 5 | 0.071 | 5.381 | 0.003 | 0.663 | 5 | 0.133 | 44.314 | 0.000 |
|  | **Within Groups** | 0.238 | 18 | 0.013 |  |  | 0.054 | 18 | 0.003 |  |  |
|  | **Total** | 0.595 | 23 |  |  |  | 0.717 | 23 |  |  |  |

**One-way ANOVA for data shown in Figure 4.**

|  | |  | **NH_4_^+^** | | | | | | | | | | |
| --- | --- | --- | --- | --- | --- | --- | --- | --- | --- | --- | --- | --- | --- |
|  | |  | **WW** | | | | | | **DS** | | | | |
|  | |  | **Sum of Squares** | | **df** | **Mean Square** | **F** | **Sig.** | **Sum of Squares** | **df** | **Mean Square** | **F** | **Sig.** |
| **Initial *A_N_*** | **Between Groups** | | | 887.4 | 5 | 177.48 | 38.292 | 0.000 |  |  |  |  |  |
|  | **Within Groups** | | | 55.619 | 12 | 4.635 |  |  |  |  |  |  |  |
|  | **Total** | | | 943.019 | 17 |  |  |  |  |  |  |  |  |
| **Initial *gs*** | **Between Groups** | | | 0.771 | 5 | 0.154 | 7.815 | 0.002 |  |  |  |  |  |
|  | **Within Groups** | | | 0.237 | 12 | 0.02 |  |  |  |  |  |  |  |
|  | **Total** | | | 1.008 | 17 |  |  |  |  |  |  |  |  |
| **14-drought *A_N_*** | **Between Groups** | | | 0.627 | 5 | 0.125 | 32.552 | 0.000 | 0.066 | 5 | 0.013 | 1.991 | 0.152 |
|  | **Within Groups** | | | 0.046 | 12 | 0.004 |  |  | 0.079 | 12 | 0.007 |  |  |
|  | **Total** | | | 0.673 | 17 |  |  |  | 0.145 | 17 |  |  |  |
| **14-drought *gs*** | **Between Groups** | | | 0.467 | 5 | 0.093 | 13.522 | 0.000 | 0.121 | 5 | 0.024 | 2.309 | 0.109 |
|  | **Within Groups** | | | 0.083 | 12 | 0.007 |  |  | 0.126 | 12 | 0.01 |  |  |
|  | **Total** | | | 0.55 | 17 |  |  |  | 0.246 | 17 |  |  |  |

|  |  | **Urea** | | | | | | | | | |
| --- | --- | --- | --- | --- | --- | --- | --- | --- | --- | --- | --- |
|  |  | **WW** | | | | | **DS** | | | | |
|  |  | Sum of Squares | df | Mean Square | F | Sig. | Sum of Squares | df | Mean Square | F | Sig. |
| **Initial *A_N_*** | Between Groups | 0.831 | 5 | 0.166 | 17.22 | 0 |  |  |  |  |  |
|  | Within Groups | 0.116 | 12 | 0.01 |  |  |  |  |  |  |  |
|  | Total | 0.947 | 17 |  |  |  |  |  |  |  |  |
| **Initial *gs*** | Between Groups | 0.83 | 5 | 0.166 | 12.649 | 0 |  |  |  |  |  |
|  | Within Groups | 0.157 | 12 | 0.013 |  |  |  |  |  |  |  |
|  | Total | 0.987 | 17 |  |  |  |  |  |  |  |  |
| **14-drought *A_N_*** | Between Groups | 0.734 | 5 | 0.147 | 26.923 | 0 | 0.561 | 5 | 0.112 | 10.845 | 0 |
|  | Within Groups | 0.065 | 12 | 0.005 |  |  | 0.124 | 12 | 0.01 |  |  |
|  | Total | 0.8 | 17 |  |  |  | 0.686 | 17 |  |  |  |
| **14-drought *gs*** | Between Groups | 0.689 | 5 | 0.138 | 10.949 | 0 | 0.519 | 5 | 0.104 | 8.52 | 0.001 |
|  | Within Groups | 0.151 | 12 | 0.013 |  |  | 0.146 | 12 | 0.012 |  |  |
|  | Total | 0.84 | 17 |  |  |  | 0.665 | 17 |  |  |  |

**One-way ANOVA for data shown in Figure 5.**

|  |  | **NH_4_^+^** | | | | | | | | | |
| --- | --- | --- | --- | --- | --- | --- | --- | --- | --- | --- | --- |
|  |  | **WW** | | | | | **DS** | | | | |
|  |  | **Sum of Squares** | **df** | **Mean Square** | **F** | **Sig.** | **Sum of Squares** | **df** | **Mean Square** | **F** | **Sig.** |
| ***ZmPIP2;4*** | **Between Groups** | 1.666 | 5 | 0.333 | 5.398 | 0.012 | 0.437 | 5 | 0.087 | 4.845 | 0.01 |
|  | **Within Groups** | 0.617 | 10 | 0.062 |  |  | 0.216 | 12 | 0.018 |  |  |
|  | **Total** | 2.283 | 15 |  |  |  | 0.653 | 17 |  |  |  |
| ***ZmTIP1;1*** | **Between Groups** | 0.986 | 5 | 0.197 | 5.573 | 0.008 | 1.685 | 5 | 0.337 | 21.024 | 0 |
|  | **Within Groups** | 0.389 | 11 | 0.035 |  |  | 0.192 | 12 | 0.016 |  |  |
|  | **Total** | 1.376 | 16 |  |  |  | 1.878 | 17 |  |  |  |
| ***ZmTIP4;1*** | **Between Groups** | 7.575 | 5 | 1.515 | 14.163 | 0.000 | 3.015 | 5 | 0.603 | 23.245 | 0 |
|  | **Within Groups** | 1.177 | 11 | 0.107 |  |  | 0.311 | 12 | 0.026 |  |  |
|  | **Total** | 8.751 | 16 |  |  |  | 3.327 | 17 |  |  |  |
| ***ZmNIP2;1*** | **Between Groups** | 0.346 | 5 | 0.069 | 5.584 | 0.008 | 1.304 | 5 | 0.261 | 6.469 | 0 |
|  | **Within Groups** | 0.136 | 11 | 0.012 |  |  | 0.484 | 12 | 0.04 |  |  |
|  | **Total** | 0.482 | 16 |  |  |  | 1.787 | 17 |  |  |  |

|  |  | **Urea** | | | | | | | | | |
| --- | --- | --- | --- | --- | --- | --- | --- | --- | --- | --- | --- |
|  |  | **WW** | | | | | **DS** | | | | |
|  |  | **Sum of Squares** | **df** | **Mean Square** | **F** | **Sig.** | **Sum of Squares** | **df** | **Mean Square** | **F** | **Sig.** |
| ***ZmPIP2;4*** | **Between Groups** | 2.838 | 5 | 0.568 | 3.611 | 0.032 | 0.248 | 5 | 0.05 | 0.749 | 0.604 |
|  | **Within Groups** | 1.887 | 12 | 0.157 |  |  | 0.729 | 11 | 0.066 |  |  |
|  | **Total** | 4.725 | 17 |  |  |  | 0.977 | 16 |  |  |  |
| ***ZmTIP1;1*** | **Between Groups** | 1.324 | 5 | 0.265 | 11.734 | 0 | 1.043 | 5 | 0.209 | 5.113 | 0.01 |
|  | **Within Groups** | 0.271 | 12 | 0.023 |  |  | 0.489 | 12 | 0.041 |  |  |
|  | **Total** | 1.595 | 17 |  |  |  | 1.532 | 17 |  |  |  |
| ***ZmTIP4;1*** | **Between Groups** | 13.698 | 5 | 2.74 | 29.596 | 0 | 5.605 | 5 | 1.121 | 3.438 | 0.046 |
|  | **Within Groups** | 0.926 | 10 | 0.093 |  |  | 3.26 | 10 | 0.326 |  |  |
|  | **Total** | 14.624 | 15 |  |  |  | 8.865 | 15 |  |  |  |
| ***ZmNIP2;1*** | **Between Groups** | 1.832 | 5 | 0.366 | 25.646 | 0 | 2.92 | 5 | 0.584 | 44.654 | 0 |
|  | **Within Groups** | 0.171 | 12 | 0.014 |  |  | 0.144 | 11 | 0.013 |  |  |
|  | **Total** | 2.003 | 17 |  |  |  | 3.064 | 16 |  |  |  |
