# Supplementary material for: Elucidating the Possible Involvement of Maize Aquaporins and Arbuscular Mycorrhizal Symbiosis in the Plant Ammonium and Urea Transport under Drought Stress Conditions
Source: Plants (Basel). 2020 Jan 23;9(2):148. doi: 10.3390/plants9020148 (PMC7076390; doi:10.3390/plants9020148)
Supplement: Supplementary file 1 [file plants-09-00148-s001.zip › supplementary/Table S2 Multifactorial_ANOVA.docx]

|  |  | **NH_4_^+^** | | | **Urea** | | |
| --- | --- | --- | --- | --- | --- | --- | --- |
|  | **Factor** | **df** | **F** | **Sig.** | **df** | **F** | **Sig.** |
| **Total FW** | **Water** | 1 | 45.06 | 0 | 1 | 34.362 | 0 |
|  | **Myc** | 1 | 74.303 | 0 | 1 | 159.121 | 0 |
|  | **N** | 2 | 1288.368 | 0 | 2 | 1474.074 | 0 |
|  | **Water * Myc** | 1 | 1.285 | 0.274 | 1 | 4.301 | 0.053 |
|  | **Water * N** | 2 | 1.837 | 0.191 | 2 | 10.198 | 0.001 |
|  | **Myc *N** | 2 | 39.07 | 0 | 2 | 55.842 | 0 |
|  | **Water * Myc * N** | 2 | 3.422 | 0.058 | 2 | 0.979 | 0.395 |
| **Mycorrhization** | **Water** | 1 | 1.998 | 0.183 | 1 | 2.03 | 0.18 |
|  | **N** | 2 | 0.074 | 0.929 | 2 | 2.22 | 0.151 |
|  | **Water * N** | 2 | 0.5 | 0.619 | 2 | 3.776 | 0.053 |
| **MDA** | **Water** | 1 | 0.652 | 0.431 | 1 | 6.48 | 0.02 |
|  | **Myc** | 1 | 10.991 | 0.004 | 1 | 9.567 | 0.006 |
|  | **N** | 2 | 22.091 | 0 | 2 | 68.182 | 0 |
|  | **Water * Myc** | 1 | 1.812 | 0.197 | 1 | 13.787 | 0.002 |
|  | **Water * N** | 2 | 0.354 | 0.707 | 2 | 1.727 | 0.206 |
|  | **Myc *N** | 2 | 10.555 | 0.001 | 2 | 0.444 | 0.648 |
|  | **Water * Myc * N** | 2 | 0.293 | 0.75 | 2 | 0.256 | 0.777 |
| **SPAD** | **Water** | 1 | 0.316 | 0.582 | 1 | 0.968 | 0.338 |
|  | **Myc** | 1 | 2.519 | 0.132 | 1 | 0.711 | 0.41 |
|  | **N** | 2 | 163.169 | 0 | 2 | 289.09 | 0 |
|  | **Water * Myc** | 1 | 0.011 | 0.916 | 1 | 7.091 | 0.016 |
|  | **Water * N** | 2 | 1.58 | 0.237 | 2 | 1.68 | 0.214 |
|  | **Myc *N** | 2 | 0.725 | 0.499 | 2 | 3.726 | 0.044 |
|  | **Water * Myc * N** | 2 | 0.055 | 0.947 |  | 1.343 | 0.286 |
| **Total N Root** | **Water** | 1 | 0.606 | 0.448 | 1 | 12.247 | 0.003 |
|  | **Myc** | 1 | 0.184 | 0.674 | 1 | 3.495 | 0.078 |
|  | **N** | 2 | 38.092 | 0 | 2 | 17.171 | 0 |
|  | **Water * Myc** | 1 | 9.311 | 0.008 | 1 | 0.017 | 0.897 |
|  | **Water * N** | 2 | 1.358 | 0.285 | 2 | 2.867 | 0.083 |
|  | **Myc *N** | 2 | 7.522 | 0.005 | 2 | 1.547 | 0.24 |
|  | **Water * Myc * N** | 2 | 8.88 | 0.003 | 2 | 0.091 | 0.913 |
| **Total NLeaves** | **Water** | 1 | 7.987 | 0.012 | 1 | 3.406 | 0.081 |
|  | **Myc** | 1 | 0.083 | 0.777 | 1 | 4.123 | 0.057 |
|  | **N** | 2 | 16.27 | 0 | 2 | 22.956 | 0 |
|  | **Water * Myc** | 1 | 0 | 0.996 | 1 | 2.397 | 0.139 |
|  | **Water * N** | 2 | 3.756 | 0.046 | 2 | 3.066 | 0.071 |
|  | **Myc *N** | 2 | 6.927 | 0.007 | 2 | 1.28 | 0.302 |
|  | **Water * Myc * N** | 2 | 0.146 | 0.865 | 2 | 0.049 | 0.952 |
| **Total C Root** | **Water** | 1 | 4.615 | 0.047 | 1 | 9.273 | 0.007 |
|  | **Myc** | 1 | 0.338 | 0.923 | 1 | 0.778 | 0.389 |
|  | **N** | 2 | 1.414 | 0.272 | 2 | 0.261 | 0.773 |
|  | **Water * Myc** | 1 | 0.377 | 0.548 | 1 | 0.065 | 0.802 |
|  | **Water * N** | 2 | 0.133 | 0.876 | 2 | 0.949 | 0.406 |
|  | **Myc *N** | 2 | 0.275 | 0.763 | 2 | 0.017 | 0.983 |
|  | **Water * Myc * N** | 2 | 0.267 | 0.769 | 2 | 0.867 | 0.437 |
| **Total C Leaves** | **Water** | 1 | 0.222 | 0.644 | 1 | 0.572 | 0.459 |
|  | **Myc** | 1 | 0.691 | 0.418 | 1 | 0.004 | 0.951 |
|  | **N** | 2 | 5.287 | 0.017 | 2 | 2.677 | 0.096 |
|  | **Water * Myc** | 1 | 0.561 | 0.465 | 1 | 1.985 | 0.176 |
|  | **Water * N** | 2 | 1.965 | 0.173 | 2 | 6.595 | 0.007 |
|  | **Myc *N** | 2 | 0.057 | 0.945 | 2 | 0.224 | 0.802 |
|  | **Water * Myc * N** | 2 | 1.942 | 0.176 | 2 | 0.653 | 0.532 |
| **C/N Roots** | **Water** | 1 | 1.466 | 0.244 | 1 | 0.239 | 0.631 |
|  | **Myc** | 1 | 0.916 | 0.353 | 2 | 11.551 | 0.001 |
|  | **N** | 2 | 10.92 | 0.001 | 1 | 0.079 | 0.783 |
|  | **Water * Myc** | 1 | 0.977 | 0.338 | 1 | 0.006 | 0.942 |
|  | **Water * N** | 2 | 1.51 | 0.251 | 2 | 3.184 | 0.065 |
|  | **Myc *N** | 2 | 3.647 | 0.05 | 2 | 0.818 | 0.457 |
|  | **Water * Myc * N** | 2 | 3.073 | 0.074 | 2 | 0.728 | 0.496 |
| **C/N Leaves** | **Water** | 1 | 8.48 | 0.01 | 1 | 3.498 | 0.078 |
|  | **Myc** | 1 | 1.01 | 0.33 | 1 | 3.933 | 0.063 |
|  | **N** | 2 | 13.095 | 0 | 2 | 23.168 | 0 |
|  | **Water * Myc** | 1 | 0.009 | 0.927 | 1 | 2.703 | 0.117 |
|  | **Water * N** | 2 | 4.326 | 0.031 | 2 | 3.09 | 0.07 |
|  | **Myc *N** | 2 | 2.506 | 0.113 | 2 | 1.264 | 0.306 |
|  | **Water * Myc * N** | 2 | 0.693 | 0.515 | 2 | 0.051 | 0.95 |
| **Initial *A_N_*** | **Myc** | 2 | 83.222 | 0 | 1 | 0.245 | 0.629 |
|  | **N** | 1 | 15.529 | 0.002 | 2 | 42.836 | 0 |
|  | **Myc *N** | 2 | 4.744 | 0.03 | 2 | 0.092 | 0.913 |
| **Initial *gs*** | **Myc** | 1 | 4.046 | 0.067 | 1 | 0.123 | 0.732 |
|  | **N** | 2 | 15.711 | 0 | 2 | 31.555 | 0 |
|  | **Myc *N** | 2 | 1.804 | 0.207 | 2 | 0.007 | 0.994 |
| **14-drought *A_N_*** | **Water** | 1 | 11.711 | 0.003 | 1 | 6.966 | 0.017 |
|  | **Myc** | 1 | 8.088 | 0.012 | 1 | 18.464 | 0 |
|  | **N** | 2 | 21.569 | 0 | 2 | 6.538 | 0.007 |
|  | **Water * Myc** | 1 | 0.2 | 0.661 | 1 | 0.768 | 0.392 |
|  | **Water * N** | 2 | 12.746 | 0 | 2 | 17.504 | 0 |
|  | **Myc *N** | 2 | 5.536 | 0.015 | 2 | 20.648 | 0 |
|  | **Water * Myc * N** | 2 | 0.904 | 0.425 | 2 | 1.055 | 0.369 |
| **14-drought *gs*** | **Water** | 1 | 7.181 | 0.016 | 1 | 4.743 | 0.043 |
|  | **Myc** | 1 | 12.808 | 0.003 | 1 | 20.52 | 0 |
|  | **N** | 2 | 5.021 | 0.02 | 2 | 1.916 | 0.176 |
|  | **Water * Myc** | 1 | 0.011 | 0.916 | 1 | 0.02 | 0.889 |
|  | **Water * N** | 2 | 13.715 | 0 | 2 | 15.26 | 0 |
|  | **Myc *N** | 2 | 3.641 | 0.05 | 2 | 13.429 | 0 |
|  | **Water * Myc * N** | 2 | 0.55 | 0.588 | 2 | 0.121 | 0.886 |
| ***ZmPIP2;4*** | **Water** | 1 | 727.258 | 0 | 1 | 1.207 | 0.286 |
|  | **Myc** | 1 | 0.793 | 0.386 | 2 | 4.121 | 0.034 |
|  | **N** | 2 | 3.073 | 0.074 | 1 | 0.902 | 0.355 |
|  | **Water * Myc** | 1 | 3.848 | 0.067 | 1 | 0.045 | 0.835 |
|  | **Water * N** | 2 | 4.233 | 0.033 | 2 | 0.162 | 0.852 |
|  | **Myc *N** | 2 | 4.984 | 0.021 | 2 | 1.733 | 0.205 |
|  | **Water * Myc * N** | 2 | 1.515 | 0.25 | 2 | 1.455 | 0.26 |
| ***ZmTIP1;1*** | **Water** | 1 | 0.185 | 0.673 | 1 | 60.548 | 0 |
|  | **Myc** | 1 | 0.026 | 0.875 | 1 | 0.076 | 0.786 |
|  | **N** | 2 | 32.65 | 0 | 2 | 21.827 | 0 |
|  | **Water * Myc** | 1 | 0.168 | 0.688 | 1 | 0.355 | 0.559 |
|  | **Water * N** | 2 | 0.705 | 0.509 | 2 | 0.554 | 0.584 |
|  | **Myc *N** | 2 | 0.278 | 0.761 | 2 | 3.149 | 0.067 |
|  | **Water * Myc * N** | 2 | 1.98 | 0.171 | 2 | 0.54 | 0.592 |
| ***ZmTIP4;1*** | **Water** | 1 | 13.35 | 0.002 | 1 | 20.748 | 0 |
|  | **Myc** | 1 | 50.482 | 0 | 1 | 17.718 | 0.001 |
|  | **N** | 2 | 34.001 | 0 | 2 | 19.586 | 0 |
|  | **Water * Myc** | 1 | 6.475 | 0.022 | 1 | 1.392 | 0.253 |
|  | **Water * N** | 2 | 0.183 | 0.835 | 2 | 5.281 | 0.016 |
|  | **Myc *N** | 2 | 3.552 | 0.053 | 2 | 4.365 | 0.028 |
|  | **Water * Myc * N** | 2 | 2.666 | 0.1 | 2 | 1.178 | 0.33 |
| ***ZmNIP2;1*** | **Water** | 1 | 1.679 | 0.213 | 1 | 121.27 | 0 |
|  | **Myc** | 1 | 1.825 | 0.196 | 1 | 37.898 | 0 |
|  | **N** | 2 | 4.428 | 0.029 | 2 | 53.432 | 0 |
|  | **Water * Myc** | 1 | 1.165 | 0.296 | 1 | 19.219 | 0 |
|  | **Water * N** | 2 | 9.619 | 0.002 | 2 | 77.538 | 0 |
|  | **Myc *N** | 2 | 3.358 | 0.061 | 2 | 3.878 | 0.04 |
|  | **Water * Myc * N** | 2 | 0.943 | 0.41 | 2 | 9.998 | 0.001 |
| **New N_Leaf** | **Water** | 1 | 5.196 | 0.032 | 1 | 10.443 | 0.004 |
|  | **Myc** | 1 | 54.725 .000 | | 1 | 52.768 | 0 |
|  | **N** | 1 | 72.137 | 0 | 1 | 78.03 | 0 |
|  | **Water * Myc** | 1 | 0.262 | 0.613 | 1 | 13.362 | 0.001 |
|  | **Water * N** | 1 | 1.546 | 0.226 | 1 | 44.466 | 0 |
|  | **Myc *N** | 1 | 93.549 | 0 | 1 | 65.267 | 0 |
|  | **Water * Myc * N** | 1 | 0.012 | 0.915 | 1 | 10.513 | 0.003 |
| **New N_Stem** | **Water** | 1 | 0.717 | 0.406 | 1 | 0.011 | 0.916 |
|  | **Myc** | 1 | 0.756 | 0.393 | 1 | 11.963 | 0.002 |
|  | **N** | 1 | 9.385 | 0.005 | 1 | 14.007 | 0.001 |
|  | **Water * Myc** | 1 | 5.15 | 0.033 | 1 | 1.032 | 0.32 |
|  | **Water * N** | 1 | 1.794 | 0.193 | 1 | 27.002 | 0 |
|  | **Myc *N** | 1 | 1.451 | 0.24 | 1 | 17.925 | 0 |
|  | **Water * Myc * N** | 1 | 0.244 | 0.626 | 1 | 9.367 | 0.005 |
| **New N_Root** | **Water** | 1 | 45.625 | 0 | 1 | 0.718 | 0.405 |
|  | **Myc** | 1 | 22.571 | 0 | 1 | 1.986 | 0.172 |
|  | **N** | 1 | 22.64 | 0 | 1 | 0.553 | 0.464 |
|  | **Water * Myc** | 1 | 4.052 | 0.055 | 1 | 0.237 | 0.631 |
|  | **Water * N** | 1 | 0.165 | 0.688 | 1 | 0.112 | 0.741 |
|  | **Myc *N** | 1 | 33.419 | 0 | 1 | 0.058 | 0.812 |
|  | **Water * Myc * N** | 1 | 2.451 | 0.131 | 1 | 6.834 | 0.015 |
